# Diagnostic performance of Oncuria™, a urinalysis test for bladder cancer

**DOI:** 10.1186/s12967-021-02796-4

**Published:** 2021-04-06

**Authors:** Yosuke Hirasawa, Ian Pagano, Runpu Chen, Yijun Sun, Yunfeng Dai, Amit Gupta, Sergei Tikhonenkov, Steve Goodison, Charles J. Rosser, Hideki Furuya

**Affiliations:** 1 Cedars-Sinai Medical Center, Samuel Oschin Comprehensive Cancer Institute, Los Angeles, CA USA; 2 Cancer Prevention and Control Program, University of Hawaii Cancer Center, Honolulu, HI USA; 3 Department of Microbiology and Immunology, The State University of New York at Buffalo, Buffalo, NY USA; 4 Department of Epidemiology, University of Florida, Gainesville, FL USA; 5 Division of Urology, Cedars-Sinai Medical Center, Los Angeles, CA USA; 6 Translational and Clinical Program, University of Hawaii Cancer Center, Honolulu, HI USA; 7 Quantitative Health Sciences, Mayo Clinic Florida, Jacksonville, FL USA; 8 Nonagen Bioscience Corp., Los Angeles, CA USA

**Keywords:** Biomarkers, Bladder cancer, Multiplex, Protein, Urinalysis

## Abstract

**Background:**

Due to insufficient accuracy, urine-based assays currently have a limited role in the management of patients with bladder cancer. The identification of multiplex molecular signatures associated with disease has the potential to address this deficiency and to assist with accurate, non-invasive diagnosis and monitoring.

**Methods:**

To evaluate the performance of Oncuria™, a multiplex immunoassay for bladder detection in voided urine samples. The test was evaluated in a multi-institutional cohort of 362 prospectively collected subjects presenting for bladder cancer evaluation. The parallel measurement of 10 biomarkers (A1AT, APOE, ANG, CA9, IL8, MMP9, MMP10, PAI1, SDC1 and VEGFA) was performed in an independent clinical laboratory. The ability of the test to identify patients harboring bladder cancer was assessed. Bladder cancer status was confirmed by cystoscopy and tissue biopsy. The association of biomarkers and demographic factors was evaluated using linear discriminant analysis (LDA) and predictive models were derived using supervised learning and cross-validation analyses. Diagnostic performance was assessed using ROC curves.

**Results:**

The combination of the 10 biomarkers provided an AUROC 0.93 [95% CI 0.87–0.98], outperforming any single biomarker. The addition of demographic data (age, sex, and race) into a hybrid signature improved the diagnostic performance AUROC 0.95 [95% CI 0.90–1.00]. The hybrid signature achieved an overall sensitivity of 0.93, specificity of 0.93, PPV of 0.65 and NPV of 0.99 for bladder cancer classification. Sensitivity values of the diagnostic panel for high-grade bladder cancer, low-grade bladder cancer, MIBC and NMIBC were 0.94, 0.89, 0.97 and 0.93, respectively.

**Conclusions:**

Urinary levels of a biomarker panel enabled the accurate discrimination of bladder cancer patients and controls. The multiplex Oncuria™ test can achieve the efficient and accurate detection and monitoring of bladder cancer in a non-invasive patient setting.

**Supplementary Information:**

The online version contains supplementary material available at 10.1186/s12967-021-02796-4.

## Background

Given the complexity of the molecular changes involved in the development of neoplastic disease, a necessary shift from the use of single diagnostic biomarkers to molecular signatures for patient evaluation has occurred. A multiplex diagnostic signature has the potential to perform accurately across the clinical and molecular spectrum of a disease, making individualized patient evaluation and care feasible. Coupled with advances in analytical instrument design, which enable the cost-effective, simultaneous measurement of molecular panels, multiplex tests are emerging as powerful tools. Several molecular signature assays have been incorporated into clinical practice for the management of prostate cancer [1, 2], breast cancer [3, 4] and colon cancer [5, 6]. However, no molecular signatures have been successfully incorporated into clinical practice for the management of bladder cancer. Bladder cancer is among the most common malignancies worldwide, and due to high rates of recurrence, one of the most prevalent.

The current primary diagnostic approach to bladder cancer is cystoscopy coupled with voided urine cytology (VUC). Cystoscopy is an uncomfortable, invasive procedure associated with significant cost and possible infection and trauma. VUC remains the method of choice for the noninvasive detection of bladder cancer. However, while the assay has good specificity, VUC sensitivity is suboptimal, especially for low-grade and low-stage tumors [7]. Consequently, the development of an accurate diagnostic bladder cancer assay that could be applied to non-invasively obtained urine samples would benefit both patients and health care systems.

**Fig. 1:**
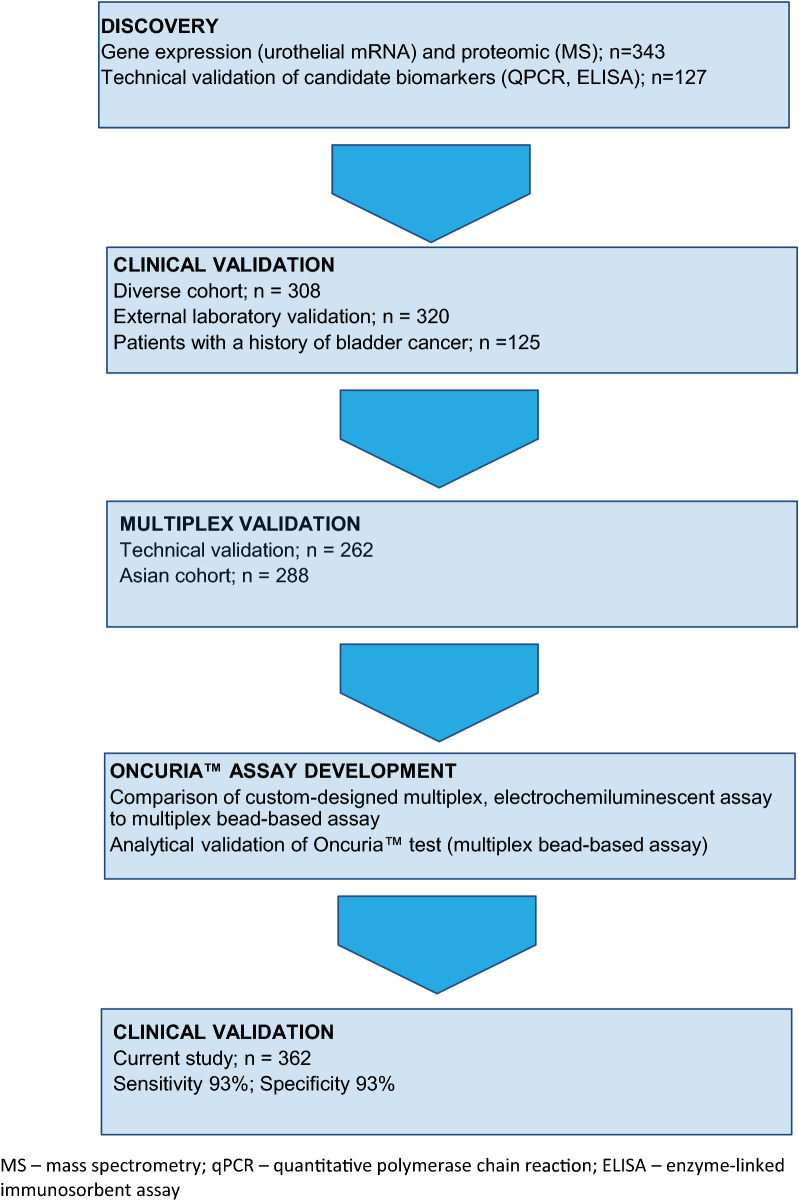
Flow diagram of phases project. Gene expression profiling (Affymetrix U133 Plus 2.0 arrays) followed by quantitative PCR verification, and glycoprotein profiling (dual-lectin affinity chromatography and liquid chromatography/tandem mass spectrometry) followed by Western blot analysis or ELISA verification were used to discover and validate RNA and protein expression profiles associated with bladder cancer. Data integration informed the selection of a 19-biomarker panel for testing which was narrowed to 10 protein biomarkers which has been validated in independent cohorts using commercial ELISA assays or custom-designed multiplex assay. The resulting Oncuria™assay used in this study has been analytically validated

In a series of previous studies, we have identified a panel of urine-based protein biomarkers that are significantly associated with bladder cancer [8–11]. The potential utility of the diagnostic panel was subsequently refined and validated in retrospective studies [12–17]. The optimal 10-biomarker panel; angiogenin, ANG; apolipoprotein E, APOE; alpha-1 antitrypsin, A1AT; carbonic anhydrase 9, CA9; interleukin 8, IL8; matrix metallopeptidase 9, MMP9; matrix metallopeptidase 10, MMP10; plasminogen activator inhibitor 1, PAI1; syndecan 1, SDC1 and vascular endothelial growth factor A, VEGFA [18]) was developed into a clinical grade, custom-designed multiplex immunoassay [19], and subsequently analytically validated, Fig. 1.

In this study, we tested the potential clinical utility of the Oncuria™ multiplex immunoassay for the detection of bladder cancer in a prospectively recruited cohort of patients who presented for urological evaluation at three institutions. The Oncuria™ test achieved a strong overall diagnostic performance, achieving an overall AUC of 0.95, sensitivity and specificity values of 93% and 93%, respectively, and a negative predictive value (NPV) and positive predictive value (PPV) of 99% and 65%, respectively. The Oncuria™ test shows promise for clinical application in the non-invasive diagnosis and surveillance bladder cancer, and potentially for screening at-risk, asymptomatic individuals.

## Methods

### Patients and specimen processing

Ethical review of the study was performed by local institutional review boards. Patients visiting the Urology outpatient clinics at University of Hawaii Cancer Center and Cedars-Sinai Medical Center were consented. As this is a urine-based assay to detect a bladder cancer associated protein signature, subjects with a history of renal insufficiency, glomerular filtration rate (GFR) < 60 mL/min and/or reduced urinary creatinine (< 40 mg/dL) were excluded, since these patients are known to have large quantities of proteins in their urine. The study cohort (Table 1) was comprised of 362 subjects, 46 de-novo bladder cancer cases and 316 non-bladder cancer controls. No one had a history of bladder cancer. Control subjects were noted to have voiding symptoms (226), urinary tract infections (17), urolithiasis (11) and hematuria (37 gross hematuria and 25 microscopic hematuria) but no pathology. Midstream voided urine sample was collected prior to any instrumentation for cytology and multiplex testing. Urines were centrifuged at 1,000*g* for 10 min and supernatant decanted and immediately frozen. Each institute processed the urines similarly. All patients underwent cystoscopy and upper tract imaging. When an abnormality was present on cystoscopy the patient underwent a formal transurethral resection of bladder tumor (TURBT) for histological confirmation of urothelial carcinoma, including grade and stage. Data are reported according to International Consensus Panel on Bladder Tumor Markers [20] and PROBE criteria [21].

**Table 1:** Demographic and clinical-pathologic characteristics of study cohorts

Variable	Value	*n*	Bladder Cancer	*n*	Non-Cancer Control	P
			N = 46		N = 316	
			*%*		*%*	
Age	18–54	4	8.7	141	44.6	**< 0.0001**
Age	55–64	12	26.1	96	30.4	**0.0000**
Age	65–74	17	37.0	47	14.9	**0.0000**
Age	75 +	13	28.3	32	10.1	**0.0000**
Sex	Female	11	23.9	79	25.1	0.86
Sex	Male	35	76.1	236	74.9	0.86
Race	White	31	67.4	70	22.2	**< 0.0001**
Race	Other	15	32.6	246	77.8	**0.0000**
Stage	0	15	34.1			
Stage	1	12	27.3			
Stage	2–3	17	38.6			
Grade	Low	9	19.6			
Grade	High	37	80.4			

Bold values indicate significant

### Multiplex immunoassay

The concentrations of the 10 proteins (A1AT, APOE, ANG, CA9, IL8, MMP9, MMP10, PAI1, SDC1 and VEGFA) were monitored using an analytically validated multiplex bead-based immunoassay (Oncuria™) from R&D Systems Inc. (Minneapolis, MN) for Luminex 200. Urine samples were passively thawed, centrifuged for 10 min × 1,000*g*. Urine samples were passively thawed and handled on ice prior to diluting twofold with R&D Assay Diluent 37. Samples, standards and controls (50 μl) were added to the 96 well plate in duplicate. The multiplex immunoassay was conducted according to the manufacturer’s instructions. A seven-point standard curve across the 4 log dynamic range of the assays was included in the current assay design. Plates were read on the Luminex® 100/200 (Luminex Corp, Austin, TX). Calibration curves were generated along with optimal fit in conjunction with Akaike’s information criteria (AIC) values [22].

#### Data analysis

A meta-cohort of 362 subjects (two missing clinical stage and one missing sex) was generated whose urine samples were analyzed in duplicate (n = 724 samples). Values were set to missing if the test–retest error was five standard deviations beyond the average test–retest error. Wald chi-square tests determined the association between each biomarker and bladder cancer. We investigated the diagnostic performance of individual biomarker for bladder cancer detection using the logistic regression analysis with bladder cancer status (yes *vs.* no) as the response variable and 10 biomarkers as the explanatory variables. Using cutoff values defined by a 50% predicted probability of disease, we defined each biomarker as either positive or negative when the biomarker was either ≥ or < the cutoff. Next, we analyzed the predictive power of the 10-biomarker molecular signature and a hybrid signature composed of the 10-biomarker molecular signature with three key demographic variables (age, sex and race) by constructing two models. For the molecular signature model, each sample is represented as a vector with 10 dimensions representing the 10 biomarkers. For the hybrid signature model, each sample is represented as 13-dimensional vectors with 10 dimensions representing the 10 biomarkers and the additional three dimensions representing the three demographic factors [23]. To compensate for the range variation between different biomarkers, we transformed the original biomarker data using log-transformation: ${\text{log}}_{10}(\text{Biomarker}+0.01)$. Then, we divided the cohort into a training (80%) and a test set. On the training set, we used the leave-one-out cross validation (LOOCV) method to estimate the parameters of a linear discriminant analysis (LDA) classifier [24, 25] and the performance of the classifier was evaluated on the test set. For performance evaluation, we calculated sensitivity, specificity, positive prediction value and negative prediction value, and a receiver operating characteristic (ROC) curve [26] was used to provide a direct view of how a prediction model functioned at different sensitivity and specificity levels. We evaluated the performance of the constructed classifiers on the test set. Statistical significance in this study was set at *p* < 0.05 and all reported *p* values were 2-sided. All analyses were performed using SAS software version 9.3 (SAS Institute Inc., Cary, NC).

## Results

The study population was comprised of 362 subjects, 287 from the University of Hawaii Cancer Center and 75 subjects from Cedars-Sinai Medical Center. Clinical, pathologic and demographic characteristics of the 362 subjects (46 bladder cancer, 316 non-bladder cancer) comprising the study cohort are listed in Table 1. Median age of bladder cancer subjects was 69 years (range 38–87 years). Of the bladder cancer subjects, 76.1% were men and 67.4% were Caucasian. Of the 46 bladder cancer cases, 61.4% were classified as non-muscle invasive bladder cancer (NMIBC; stages Ta, Tis, T1), and 38.6% were muscle invasive bladder cancer (MIBC; stage ≥ T2), while 19.6% cases were reported as low-grade carcinoma and 80.4% cases as high-grade.

To reduce skewness when comparing results from different institutes, we used the log transformation, log_10_(data + 0.01) for each biomarker. There was limited variability observed in each biomarker concentration ranges between institutions (*Supplemental Table*). Urinary concentrations of all 10 biomarkers were elevated in patients with bladder cancer compared with non-bladder cancer (Table 2) with statistical significance being reached for MMP9, IL8, VEGFA, PAI1, ApoE, A1AT and ANG.

**Table 2:** Mean urinary (± SD) concentrations of 10 biomarkers assessed by Oncuria™ in cohort of 362 subjects

Biomarker pg/mL	Detectable %	Mean	Bladder Cancer	Mean	Non-Cancer Control	P
			N = 46		N = 316	
			SD		SD	
MMP9	64.3	1,237.2	2,191.7	143.0	1,304.3	**0.002**
CXCL8/IL8	84.4	681.0	1,376.4	90.3	582.5	**0.006**
VEGFA	88.6	1,003.9	2,743.3	127.8	261.3	**0.04**
IX/CA9	40.6	8,979	35,518	0.843	2.016	0.09
SDC1	99.3	9,461	6,415	8,707	4,455	0.44
PAI1	71.7	1,169.8	2,803.0	29.8	132.9	**0.009**
ApoE	95.7	16,627	35,895	1,014	2,001	**0.005**
A1AT	93.2	179,562	236,921	33,742	67,463	**0.0001**
ANG	81.8	1,800.4	3,170.3	194.7	464.7	**0.001**
MMP10	57.7	52.79	200.47	4.92	8.88	0.12

Bold values indicate significant

Table 3 provides AUC data for each individual biomarker and the combination of the ten biomarkers and the hybrid signature. The hybrid signature achieved superior AUC values. All ten biomarkers using optimal cutoff values defined by a 50% predicted probability of disease resulted in an AUC of 0.93 (95% confidence interval, 0.87–0.98), with a sensitivity of 87%, a specificity of 92%, a negative predictive value of 98% and a positive predictive value of 61% (Table 3). The AUC improved to 0.95 (95% confidence interval, 0.90–1.00) with the addition of the three demographic factors in the hybrid signature with corresponding sensitivity of 93%, specificity of 93%, negative predictive value of 99% and positive predictive value of 65% (Table 3). Univariate analysis indicated age, race, MMP9, IL8, VEGFA, CA9, PAI1, ApoE, A1AT, ANG and MMP10 were associated with bladder cancer (Table 4).

**Table 3:** Performance of Oncuria™ for bladder cancer detection

Biomarker	Area under the curve	95% Confidence interval	No. of correctly predicted events	No. of correctly predicted nonevents	No. of nonevents predicted as events	No. of events predicted as nonevents	Sen	Spec	PPV	NPV
MMP9	0.78	[0.70 0.86]	30	267	48	15	0.67	0.85	0.38	0.95
CXCL8/IL8	0.82	[0.76 0.88]	33	256	59	12	0.73	0.81	0.36	0.96
VEGFA	0.71	[0.64 0.79]	39	156	159	6	0.87	0.50	0.20	0.96
IX/CA9	0.76	[0.69 0.83]	26	268	47	19	0.58	0.85	0.36	0.93
SDC1	0.55	[0.44 0.66]	45	37	278	0	1.00	0.12	0.14	1.00
PAI1	0.89	[0.83 0.95]	35	283	32	10	0.78	0.90	0.52	0.97
ApoE	0.89	[0.84 0.94]	33	282	33	12	0.73	0.90	0.50	0.96
A1AT	0.82	[0.76 0.88]	33	241	74	13	0.73	0.77	0.31	0.95
ANG	0.84	[0.78 0.90]	38	222	93	7	0.84	0.70	0.29	0.97
MMP10	0.73	[0.64 0.82]	24	292	23	21	0.53	0.93	0.51	0.93
10-biomarker combination	0.93	[0.87 0.98]	39	290	25	6	0.87	0.92	0.61	0.98
10-biomarker combination + 3 demographic features	0.95	[0.90 1.00]	42	292	23	3	0.93	0.93	0.65	0.99

**Table 4:** Univariate analysis of the three demographic factors and 10 biomarkers in cancers and controls

Variable	Value	Total N	Column %	Cancer N	Row %	OR	LCL	UCL	P
Age	18–54	145	40.1	4	2.8	0.07	0.02	0.23	**< 0.0001**
Age	55–64	108	29.8	12	11.1	0.31	0.13	0.74	**0.009**
Age	65–74	64	17.7	17	26.6	0.89	0.38	2.08	0.79
Age	75 +	45	12.4	13	28.9	1.00			
Sex	Female	90	24.9	11	12.2	0.94	0.46	1.94	0.86
Sex	Male	271	75.1	35	12.9	1.00			
Race	White	101	27.9	31	30.7	7.26	3.71	14.21	**< 0.0001**
Race	Other	261	72.1	15	5.7	1.00			
MMP9 (pg/mL)	< 4,681.7	351	98.3	41	11.7	1.00			
M	≥ 4,681.7	6	1.7	5	83.3	37.80	4.31	331.63	**0.001**
CXCL8/IL8 (pg/mL)	< 3,166.3	355	98.3	42	11.8	1.00			
	≥ 3,166.3	6	1.7	4	66.7	14.90	2.65	83.87	**0.002**
VEGFA (pg/mL)	< 1,837.5	350	98.3	41	11.7	1.00			
	≥ 1,837.5	6	1.7	5	83.3	37.68	4.30	330.57	**0.001**
IX/CA9 (pg/mL)	< 22.429	351	97.5	38	10.8	1.00			
	≥ 22.429	9	2.5	8	88.9	65.89	8.02	541.31	**0.0001**
SDC1 (pg/mL)	< 70,803	357	100.0	46	12.9	1.00			
	≥ 70,803	0	0.0						
PAI1 (pg/mL)	< 643.76	343	95.5	33	9.6	1.00			
	≥ 643.76	16	4.5	12	75.0	28.18	8.60	92.37	**< 0.0001**
ApoE (pg/mL)	< 9,433.5	339	95.0	31	9.1	1.00			
	≥ 9,433.5	18	5.0	15	83.3	49.68	13.63	181.09	**< 0.0001**
A1AT (pg/mL)	< 337,795	343	96.6	37	10.8	1.00			
	≥ 337,795	12	3.4	9	75.0	24.81	6.43	95.75	**<0 .0001**
ANG (pg/mL)	< 1,951.8	339	95.2	34	10.0	1.00			
	≥ 1,951.8	17	4.8	11	64.7	16.44	5.72	47.27	**<0 .0001**
MM10 (pg/mL)	< 40.975	347	97.2	38	11.0	1.00			
	≥ 40.975	10	2.8	7	70.0	18.97	4.71	76.46	**< 0.0001**

Bold values indicate significant

Urinary cytology was available in 35 of the cancer subjects with 8 being called positive (sensitivity of 22.8%). Table 5 denotes the overall sensitivity and specificity achieved using the Oncuria™ hybrid signature for low grade and high grade, and non-muscle invasive bladder cancers and muscle invasive bladder cancers.

**Table 5:** Summary of diagnostic performance of Oncuria™ in high-grade/low-grade and high stage/low stage bladder cancer

	Number of bladder cancer cases predicted by biomarker assay	AUC	Sensitivity (%)	Specificity (%)	NPV (%)	PPV (%)
Overall	42/45^a^	0.95	0.93	0.93	0.99	0.65
Low-grade tumors	8/9	0.94	0.89	0.93	1.00	0.26
High-grade tumors	34/36	0.95	0.94	0.93	1.00	0.60
NMIBC	25/27	0.93	0.93	0.93	0.99	0.52
MIBC	15/16	0.97	0.94	0.93	1.00	0.39

*NMIBC* non-muscle invasive bladder cancer, *MIBC* muscle invasive bladder cancer, *AUC* Area under ROC curve;

^a^1 missing an analyte and thus excluded

## Discussion

Cancer of the urinary bladder is a common neoplastic disease with high rates of recurrence and progression. The rate of recurrence makes it one of the most prevalent cancers worldwide [27]. Disease detection currently relies upon invasive cystoscopic examination of the bladder. The only urinary assay in routine use is voided urine cytology (VUC), but as it lacks sensitivity, it is typically deployed as an adjunct to cystoscopy rather than a stand-alone test. The development of accurate, non-invasive urinary tests would benefit both patients and health care systems. A robust test could avoid unnecessary invasive patient evaluation and improve patient compliance on clinical surveillance and follow-up regimes. The development of multiplex assays that reflect the complexity of molecular events involved in neoplasia can provide a more accurate assessment with broad clinical utility.

Multiplex assay advantages include reduced cost through lower labor needs and reagent consumption, and the generation of more data with less sample, but the major advantage is the potential to significantly improve clinical test sensitivity and specificity by a combination of multiple biomarkers. Many tissue-based analyses focus on multiplexing nucleic acid targets, but for liquid biopsy settings protein multiplexing may be more appropriate as the test is relatively straightforward with minimal sample processing, fast and economical throughput, and can achieve direct quantitation without requiring molecular target amplification. Notably, one multiplex protein cancer diagnostic test is FDA approved, OVA1, which is being employed for the early detection of ovarian cancer [28]. The test measures absolute serum levels of CA125, apolipoprotein A1, beta 2 microglobulin, prealbumin, and transferrin to determine the risk for malignancy. The test has a reported overall sensitivity of > 90% as a stand-alone test and can provide a valuable adjunct to ultrasound imaging and physical examination [28]. Coupling the advantages of a multiplex protein test with non-invasive urine sampling could provide a highly accurate bladder cancer diagnostic test as well as providing data for monitoring disease progression and response to therapy. The development of the Oncuria™ test has been reported from transcriptomic and proteomic profiling discovery [8–11], to refinement and validation of candidate biomarkers [12–15], to custom multiplex design and analytical validation [18, 19]. In this study, the test was applied to 348 naturally micturated urine samples prospectively obtained from patients visiting urology clinics at three institutions.

The 10 biomarkers associated with Oncuria™ were reliably detect in the 362 urine samples; MMP9 in 64.3%, IL8 in 84.4%, VEGFA in 88.6%, CA9 in 40.6%, SDC1 in 99.3%, PAI1 in 71.7%, ApoE in 95.7%, A1AT in 93.2%, ANG in 81.8% and MMP10 in 57.7%. Further, these 10 biomarkers were present at higher levels in voided urines from bladder cancer subjects compared to controls with significance being reached for IL8, VEGFA, PAI1, ApoE, A1AT and ANG. SDC1 had only slightly elevated mean levels in cancer compared to controls; 9,461 pg/mL vs. 8,707 pg/mL. Previously we reported that SDC1 levels are lower in controls compared to bladder cancer, but it hold prognostic significance in the high-grade and high stage tumors shed less SDC1 in voided urines than low-grade and low stage tumors [29]. Despite this, SDC1 adds value to the signature and thus is included in the combinatorial analysis of all ten biomarkers, obviously with a different trajectory in its weight compared to the other analtyes. Single biomarkers were noted to have lower sensitivity and/or specificity; best response PAI1 AUROC of 0.89 (95% confidence interval, 0.83–0.95) with a sensitivity of 78% and a specificity of 90% and ApoE AUROC of 0.89 (95% confidence interval, 0.84–0.94), with a sensitivity of 73% and a specificity of 90%. A combinatorial analysis of all ten biomarkers noted an AUROC of 0.93 (95% confidence interval, 0.87–0.98), with a sensitivity of 87% and a specificity of 92%. These parameters were noted to improve with the addition of the three demographic factors (age, sex and race) to the hybrid signature: AUROC of 0.95 (95% confidence interval, 0.90–1.00), with a sensitivity of 93% and a specificity of 93%. Lastly, we noted that urine samples from patients with history of renal cell carcinoma or renal cell carcinoma and urine samples from patients with history of prostate cancer or prostate cancer did not result in positive Oncuria™ test (*data not shown*). This finding confirms our previous report in that thus attesting to its specificity. We were able to confirm the clinical utility of monitoring a diagnostic biomarker signature for the detection of bladder cancer in non-invasively obtained urine samples. The Oncuria™ test achieved encouraging values of sensitivity and specificity and NPV.

Recently, several groups have begun to identify panels of diagnostic biomarkers for potential bladder cancer application. For example, through analysis of nine gene promoters, Hoque et al. found that 69% of bladder cancer patients had methylation in at least one of four genes (CDKN2A, ARF, MGMT, GSTP1), whereas the controls had no such methylation detectable [30]. By combining the data from all nine genes, a logistic prediction model was derived that achieved a sensitivity of 82% and specificity of 96%. Chung et al. selected 10 candidate hypermethylated genes from data collected from tumor tissue and tested these 10 genes in voided urine samples by quantitative methylation-specific RT-PCR and identified a multigene predictive model comprised of five target genes (*MYO3A, CA10, NKX6-2, DBC1*, and *SOX11*). Sensitivity and specificity of this model were 85% and 95%, respectively [31]. Further examples include RNA signatures proposed by Hanke et al. [32] and Mengual et al. [33] possessing sensitivities ranging from 80% to 92% and specificities ranging from 85% to 99%. To date, these studies have had small sample size, with limited populations analyzed (*i.e.,* few benign confounding conditions included) and have not undergone extensive validation. Only Holyoake et al. from New Zealand have reported on the discovery [34] and validation of a multiplexed RNA signature comprised of *CDC2, MDK, IGFBP5* and *HOXA13* (Cxbladder™), with a reported sensitivity of 82% and specificity of 85% [35, 36].

We recognize that the study has several limitations. First, as tertiary-care facilities, we tend to see more high-grade, high-stage disease, which is reflected in our study cohort. To further confirm the robustness of the multiplex assay, subsequent studies must assess larger cohorts that include more subjects with low-grade, low-stage disease. Second, we did not have complete smoking data for all subjects in the cohort and, therefore, an association with smoking history was not possible. Third, processed, banked urines were analyzed. Urines were centrifuged and separated into cellular pellet and supernatant before storage at – 80 °C. It is feasible that freshly voided urine samples may provide different results. We are currently investigating the performance of the test in urines processed via a number of different protocols, including freshly voided urines. To address these issues, the Oncuria™ test is currently being evaluated in three large multicenter, international prospective clinical trials (NCT 03,193,515, 03,193,528, and 03,193,541). These trials will include first-event diagnosis and disease recurrence monitoring.

## Conclusions

Bladder cancer is a common neoplastic disease encountered worldwide. The development of an accurate and robust urinary test for the detection of bladder cancer would benefit both patients and healthcare systems. In a multi-institutional cohort study, the multiplex Oncuria™ test achieved highly encouraging diagnostic performance. The test uses established technology enabling rapid uptake in clinical laboratories around the world. Additional studies are underway to evaluate the potential added value of the test in clinical decision making.

## Supplementary Information

**Additional file 1: **Boxplot of mean ± SD of urine concentrations of the 10 protein biomarkers between the bladder cancer and non-cancer groups from the participating institutes.

## Data Availability

Reasonable requests for data will be made available for review.
