# Supplementary figures and images for: Diagnostic performance of Oncuria™, a urinalysis test for bladder cancer
Source: J Transl Med. 2021 Apr 6;19:141. doi: 10.1186/s12967-021-02796-4 (PMC8025333; doi:10.1186/s12967-021-02796-4)

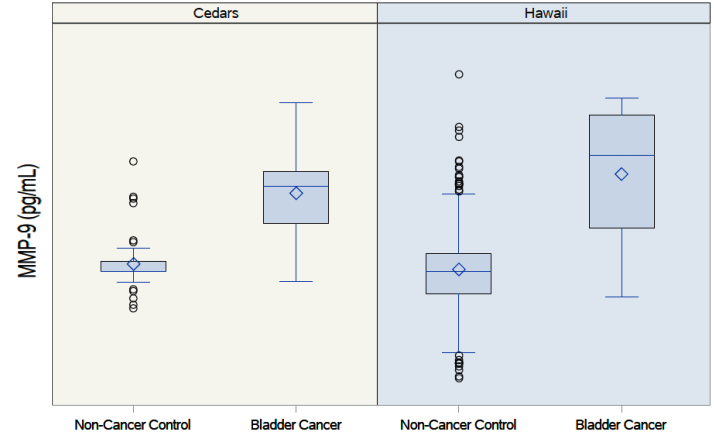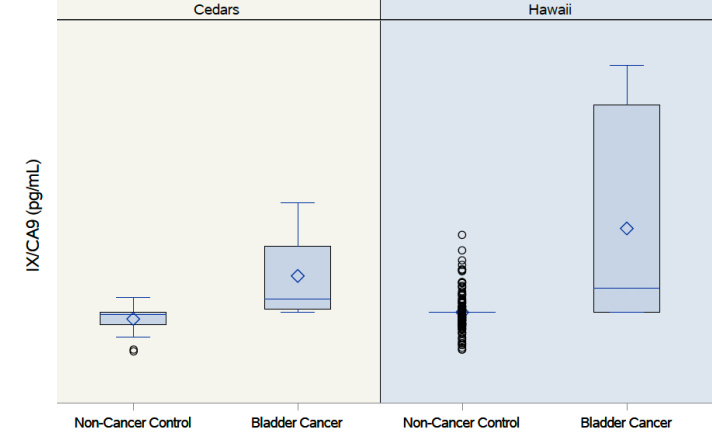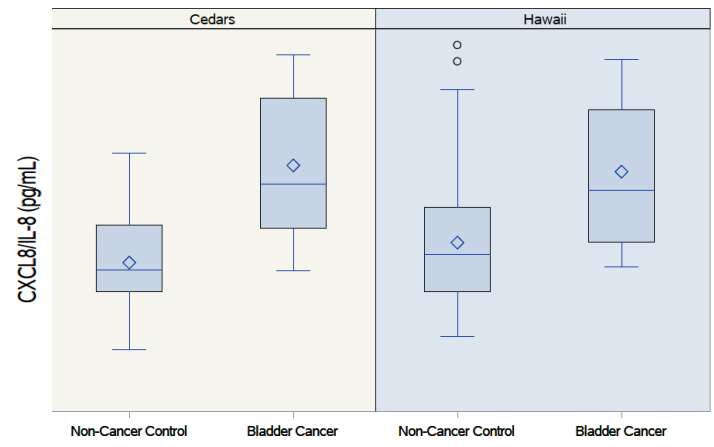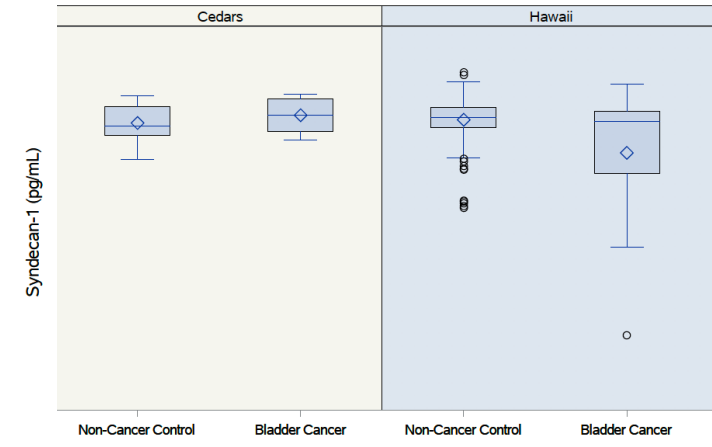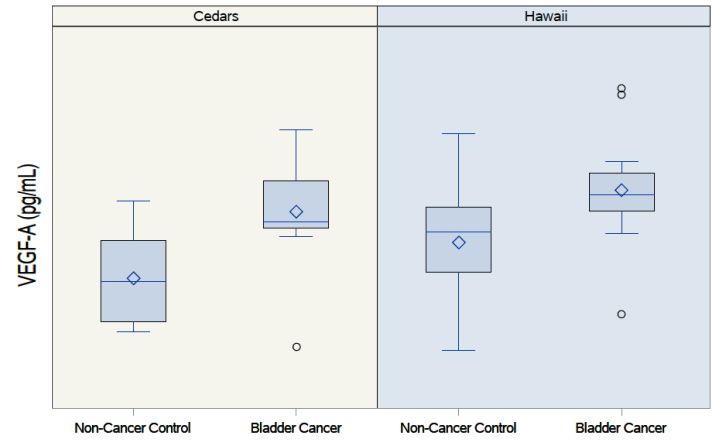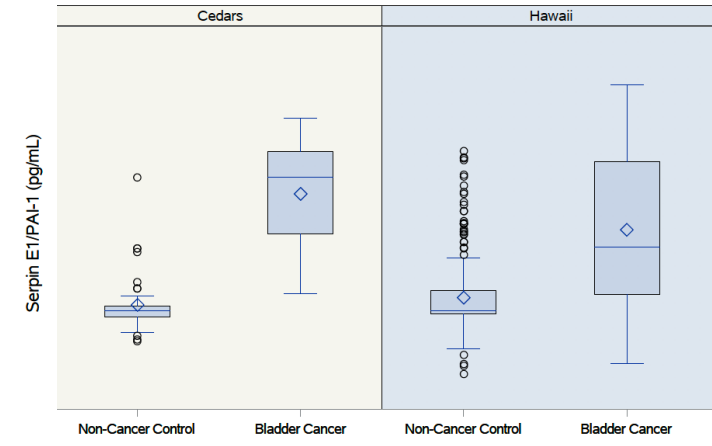

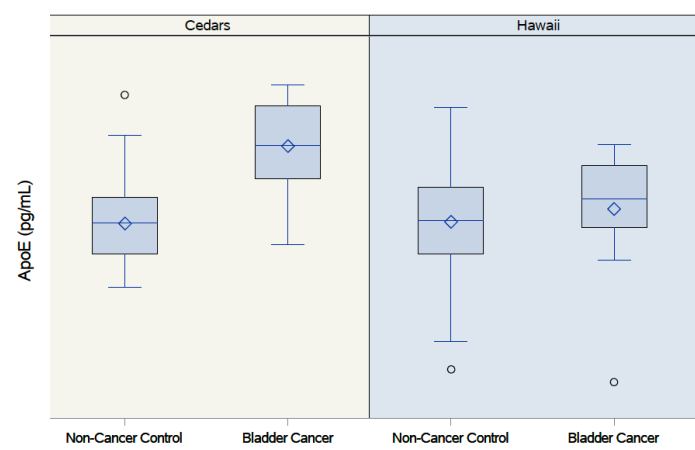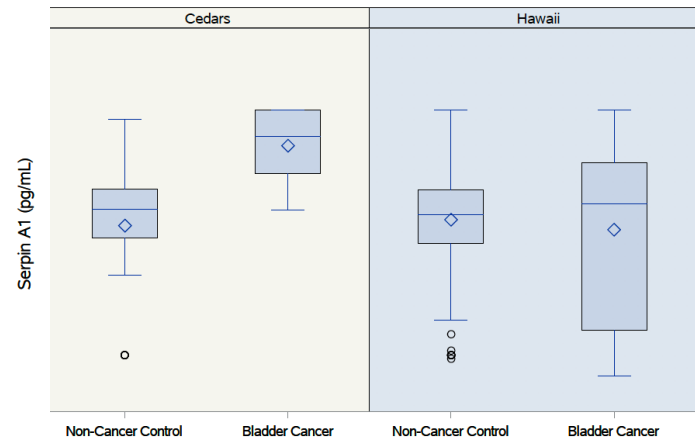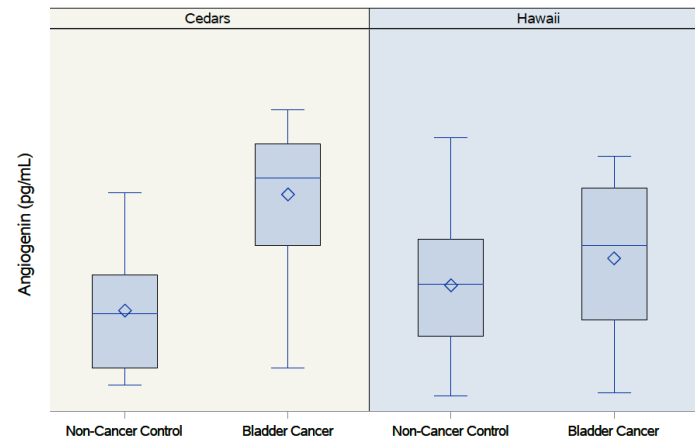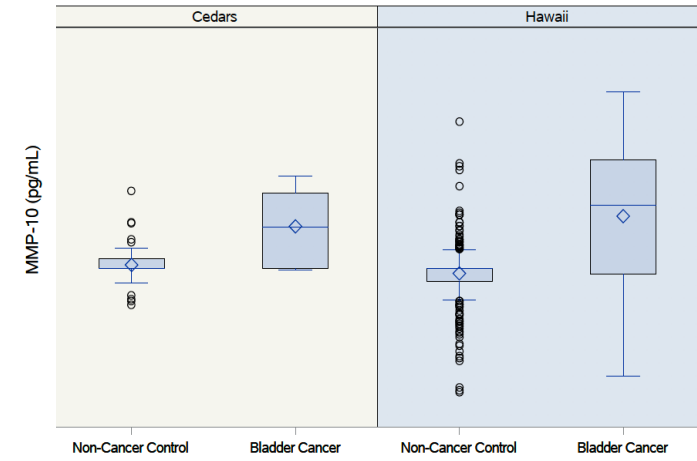

Supplement: Supplementary file 1 — Additional file 1: Boxplot of mean ± SD of urine concentrations of the 10 protein biomarkers between the bladder cancer and non-cancer groups from the participating institutes. [file 12967_2021_2796_MOESM1_ESM.pdf]
